# Impact of HIV status and predictors of successful treatment outcomes among tuberculosis patients: A six-year retrospective cohort study

**DOI:** 10.1016/j.amsu.2020.11.032

**Published:** 2020-11-15

**Authors:** Ginenus Fekadu, Ebisa Turi, Tinsae Kasu, Firomsa Bekele, Legese Chelkeba, Tadesse Tolossa, Busha Gamachu Labata, Dinka Dugassa, Getahun Fetensa, Dereje Chala Diriba

**Affiliations:** a School of Pharmacy, Institute of Health Sciences, Wollega University, Nekemte, Ethiopia; b Department of Public Health, Institute of Health Sciences, Wollega University, Nekemte, Ethiopia; c Department of Production, Julphar Pharmaceuticals PLC, Addis Ababa, Ethiopia; d Department of Pharmacy, College of Health Sciences, Mettu University, Mettu, Ethiopia; e School of Pharmacy, College of Health Sciences, Addis Ababa University, Addis Ababa, Ethiopia; f School of Nursing and Midwifery, Institute of Health Sciences, Wollega University, Nekemte, Ethiopia

**Keywords:** Predictor, TB-HIV Co-Infection, Treatment success rate, TB treatment Outcome, Ethiopia

## Abstract

Tuberculosis (TB) remains a major global public health problem. Hence, the study aimed to assess the impact of human immune virus (HIV) status and predictors of successful treatment outcomes of TB patients enrolled at Nekemte specialized hospital. An institution-based retrospective cohort study was conducted and the data analyzed using SPSS version 24.0. A multivariable logistic regression model was fitted to identify the association between treatment outcome and potential predictor variables. The association was calculated using the Adjusted Odds ratio (AOR) and the statistical significance was considered at p < 0.05. Out of the total 506 study participants, 50.2% of them were males. The overall treatment success rate was 81.4% and 58.06% among HIV co-infected TB patients. Female sex (AOR = 2.01, 95%CI: 1.04–16.11), age 25–34 years (AOR = 3.982, 95%CI: 1.445–10.971), age 35–49 years (AOR = 5.392, 95%CI: 1.674–17.368), high school educational level (AOR = 5.330, 95% CI: 1.753–16.209), urban residence (AOR = 3.093, 95%CI: 1.003–9.541) and HIV negative (AOR = 10.3, 95%CI, 3.216–32.968) were positively associated with favorable TB treatment outcome. Whereas, being single (AOR = 0.293, 95%CI: 0.1–0.854), smear-negative pulmonary TB (AOR = 0.360, 95%CI: 0.156–0.834), extra-pulmonary TB (AOR = 0.839, 95%CI: 0.560–0.955) and retreatment case (AOR: 0.54, 95%CI: 0.004–0.098) were negatively associated with successful treatment outcome. The treatment success rate of TB patients was lower than World Health Organization target set of 85%. The increased unsuccessful outcome among TB/HIV patients requires urgent public health interventions to improve the evaluation policy and control framework.

## Highlights

• Tuberculosis (TB) remains a major global public health problem.
• TB is the leading cause of death for HIV-infected patients, and HIV is also risk factor for developing active TB.
• The co-infection has emerged as a major public health threat throughout the world and have worse treatment outcomes.
• Management of co-infected patients can be complex because of overlapping drug toxicities and interactions.
• The increased unsuccessful outcome among TB/HIV patients requires urgent public health interventions.

## 1 Introduction

Tuberculosis (TB) remains a major global public health problem [[1], [2], [3], [4]]. It is the leading cause of morbidity and mortality in the 21st century [3,[5], [6], [7], [8]]. It causes illness among millions of people each year and the second leading cause of death from an infectious disease worldwide, next to immunodeficiency virus (HIV) infection [2,4,9,10]. Despite the availability of effective diagnostic tools and treatment, it has been affecting the lives and well-being of a large number of people [9,11,12].

Globally, an estimated 10 million people were infected with TB as of 2018. There were an estimated 1.2 million TB deaths among HIV-negative people in 2018 and an additional 251 000 deaths among HIV positive people [7]. TB is the leading cause of death for HIV-infected patients, and HIV is the most important risk factor for developing active TB [13]. The co-infection has emerged as a major public health threat throughout the world compared to patients with TB alone [[14], [15], [16], [17], [18]]. The risk of mortality from TB is significantly higher in the HIV-infected population [13,[19], [20], [21], [22]] due to the shared immune defense mechanisms and HIV has also contributed to a worldwide resurgence of TB [[23], [24], [25], [26]].

The clinical burden of HIV patients was worsened by the concomitant infection of TB which becomes major global health threats [17,18,27]. Untreated TB accelerates the progression of HIV infection to AIDS [21]. This is due to the HIV epidemic has greatly altered the epidemiological course of TB and modifies the natural history [22,28,29]. On the other hand, HIV infection predisposes to the development of active TB, and the course of HIV-related immunodeficiency is worsened by active TB infection [13,[30], [31], [32]]. HIV fuels the TB epidemic, and increasing the likelihood of death [6,11]. TB-HIV co-infected people are experiencing “double trouble” that puts them at high risk of mortality and rapid disease progression [27].

Management of co-infected patients can be complex because of overlapping drug toxicities and interactions [12,33]. Furthermore, HIV-positive TB patients are a challenge to TB services, as they are more likely to have diagnostic delays [[32], [33], [34], [35]]. Duration of treatment, frequency of drug administration, pill burden, and complications of therapy are some of the challenges associated with the coinfection [12,18]. TB patients who were co-infected with HIV encounter different problems in the management of their disease like high loss to follow up rates, non-adherence and relapse rate [11,36,37]. Co-infection adversely affects socio-economic development and challenges treatment outcome [6,15]. The situation is worsened by the growing cases of multidrug-resistant TB and the changing prevalence of HIV epidemics [23]. Early diagnosis and effective treatment of active TB disease in HIV-infected patients is imperative for curing TB and decreasing its ill effects [38]. Administration of concurrent ART and anti-TB therapy reduces the risk of death among TB/HIV co-infected patients [21]. But drug-drug interactions between HIV and TB therapy are common [12].

The devastating effects of TB and HIV/AIDS to health and human development remains a major public health challenge in developing countries [11,12,15,33,[39], [40], [41]], particularly in sub-Saharan Africa (SSA) [6,9,17,[42], [43], [44]]. About 80% of the total estimated disease burden of HIV associated TB is found in countries of SSA, and this part of the world has the “highest rates of cases and deaths per capita” attributable to TB disease [44]. The incidence of TB has been increasing steadily since the 1990s, particularly in African countries [45], and an estimated 30% of HIV infected persons have a dual infection with TB in SSA countries [44]. In 2015, about 75% of TB/HIV deaths worldwide occurred in SSA, with case fatality rates varying from under 5% to over 20% among countries in the region [2,21]. Regional and global profiles of the World Health Organization (WHO) of 2019 estimates of TB burden in Africa region has shown that the rate of total TB incidence was 231 per 100 000 population and HIV-positive TB incidence was 58 per 100, 000 populations [7].

The joint effect of HIV and TB pestilences has confronted the feeble systems of healthcare in resource-limited countries [9,29]. In these resource-poor settings, limited data exist both on treatment outcomes and ways to carry out interventions. As a result, the existing treatment guidelines and recommendations are based on data from the developed world [19].

The WHO and the National Tuberculosis and Leprosy Control Programme (NTBLCP) recommended a case detection rate of 70% and a treatment success rate (TSR) of 85% for all TB cases [41]. Interventions are necessary to reduce deaths during TB treatment for the country to achieve the targets set in the End TB Strategy by 2035 [21]. The integration of TB-HIV collaborative activities will help to reduce mortality, default, relapse, and drug resistance [12,31,40]. The therapeutic goals for tuberculosis aim at curing the patient, avoiding transmission, and preventing multi-drug resistant TB [2,11]. Even though TB is treatable and curable, it has proven difficult to eliminate, and this has been worsened by the HIV-AIDS pandemic [41].

TB-HIV co-infection remained a serious health problem in Ethiopia, where the disease is the leading cause of mortality and morbidity [8,15,25,37,46,47]. Regional and global profiles of WHO estimates of TB burden in Ethiopia shown the rate of total TB incidence was 151 per 100 000 population. The estimated epidemiological burden of TB in 2018 revealed that Ethiopia ranked 7th among 20 high TB burden countries based on an absolute number of incident cases and one of 10 high TB burden countries based on the severity of disease burden (incidence per capita) [7].

Despite the availability of free ART and the implementation of different strategies, the burden, and mortality of TB-HIV co-infected patients remains a challenge in Ethiopia [6,17]. Assessing TB treatment outcomes and understanding the specific reasons for unfavorable treatment outcomes is important for stakeholders working to evaluate the performance of TB treatment and to improve the management of TB-HIV co-infected patients [1,[17], [18], [19],^37^,^48^]. Hence, the study aimed to assess the impact of HIV status and predictors of successful treatment outcomes among TB patients enrolled to Nekemte specialized hospital, western Ethiopia.

## 2 Materials and methods

### 2.1 Study area and period

The study was conducted at Nekemte Specialized Hospital (NSH), western Ethiopia which is found 328 km from Addis Ababa. NSH has different departments and wards like outpatient department, medical ward, gynecology and obstetrics ward, pediatrics ward, and surgical ward. It delivers diversified health services and clinics including the emergency services, eye clinic, dental clinic, mother and child health (MCH), orthopedics, psychiatry clinic, laboratory service, imaging's, pharmacy, physiotherapy and follow up of chronic disease including TB. It has been serving TB patients as a separate TB clinic since 2013. Six years from 2013 to 2019 TB patient's treatment outcomes were reviewed from March to April 2019. The work has been reported in line with the strengthening the reporting of cohort studies in surgery (STROCSS) criteria [49].

### 2.2 Study design

A retrospective cohort study was conducted among TB patients enrolled to Nekemte Specialized Hospital (NSH). A six-year retrospective document review of TB patients registered at the Directly Observed Therapy (DOTS) was conducted to assess the impact of HIV and predictors of successful treatment outcomes among TB patients. The clinic registers and treats patients diagnosed with TB using the DOT strategy designed by the National Tuberculosis and Leprosy Control Program (NTLCP) of Ethiopia which is a guideline adapted from WHO [40,46]. The diagnosis of pulmonary TB is followed by an examination of three-morning sputum smears by Zihel –Nielsen staining method for acid-fast bacilli (AFB). Chest radiographs and pathological investigations are also used to support the diagnosis. A clinical diagnosis of TB was based on a history of persistent cough for three weeks or more and not responding to conventional antibiotics. Additionally, the diagnosis of TB-HIV coinfected patients is as per the guideline Federal Ministry of Health of Ethiopia: Manual of Tuberculosis, Leprosy and TB/HIV prevention and control program [46,50].

TB DOTS clinic of NSH serves both adult and pediatric age groups with free drugs like Rifampicin, Isoniazid, Pyrazinamide and Ethambutol (2RHZE) for 2 months' intensive phase with daily observation, and Rifampicin and Isoniazid (4RH) for 4 months continuous phase with monthly follow up for new TB patient. The hospital also provides Rifampicin, Isoniazide, Pyrazinamide, Ethambutol and Streptomycin (3RHZES) for 3 months' intensive phase and Rifampicin, Isoniazide and Ethambutol (5RHE) for 5 months' continuous phase for retreatment patients. For TB/HIV co-infected patients; they should take daily for 6 months; 2 months' intensive phase taking Rifampicin, Isoniazid, Pyrazinamide and Ethambutol (2RHZE) and 4 months’ continuation phase taking Rifampicin and Isoniazid (4RH).

All TB patients under treatment follow up were tested for HIV. For HIV screening, nationally approved rapid serological testing algorisms (KHB → StatPak → Uni-gold) were used. As soon as HIV is identified in a TB patient, the patient is enrolled to ART clinic of NSH. The national TB/HIV implementation guideline recommends provision of ART for TB/HIV co-infected individuals if they are WHO clinical stage IV or CD4 < 350/mm.

During the 2 months’ intensive phase of treatment, patients come directly to the hospital daily (directly observed therapy) and take their medication under the supervision of the health care provider in the TB clinic. During continuous phase patients followed weekly, every two week or monthly basis.

### 2.3 Population

The target population for this study was all patients diagnosed with active TB disease and were on anti-TB treatment. The study population included all new TB patients aged ≥15 years registered from 2013 TO 2019 at NSH DOTs clinic of NSH within the study period and fulfilled the inclusion criteria.

### 2.4 Eligibility criteria

#### 2.4.1 Inclusion criteria

• All adult (≥15 years old) TB patients diagnosed with active TB disease.
• Patients who have been started on a course of anti-TB treatment regimen within the time frame of the study period.

#### 2.4.2 Exclusion criteria

• Patients with multi-drug resistant TB (MDR-TB).
• Patients who were transferred to another health facility to continue their treatment.
• Adults with incomplete registration cards (like basic sociodemographic information, treatment outcome unknown)
• Patients who started anti-TB from other health care institutions (transfer-in).
• Any individual who has taken less than 4 weeks of the course of anti-TB treatment regimen (dropouts).

### 2.5 Study variables

#### 2.5.1 Dependent variable

• TB treatment outcomes: successful outcome (cured, treatment completed) and unsuccessful outcome (treatment failure, died and default)

#### 2.5.2 Independent variables

• Sociodemographic factors (age, sex, marital status, residence, educational status).
• Clinical conditions: (category of TB, TB enrollment, HIV status, WHO HIV staging, CD4 count, opportunistic infections, functional status, nutritional status, presence of another comorbidity)
• Drug-related factors (ART, Anti-TB, Isoniazid prophylactic therapy, Cotrimoxazole preventive therapy, adverse effects due to treatment).

### 2.6 Sample size and sampling technique

The sample size required for achieving statistically significant results was determined using two population proportion formula using Epi Info 7 statistical software package. Based on HIV status, TB patients were categorized into “HIV positive” and “HIV negative” cohorts and were retrospectively followed until the time of the outcome. HIV status was chosen as the main exposure variable of non-accidental mortality during the 6 years of follow up since it was considered to give the optimal sample size. In this regard, a 5% level of significance (two-sided), a power of 80% and a ratio of exposed to unexposed of 1:2.13, estimated proportion of mortality (one of the unsuccessful outcome factor) in Ethiopia was taken as 2.5% for the non-exposed group (not co-infected with HIV) and 8.3% for the exposed group (co-infected with HIV) [44]. Thus, the sample size was calculated to be 185 for exposed and 394 for non-exposed, a total of 597 patients.

**Fig. 1:**
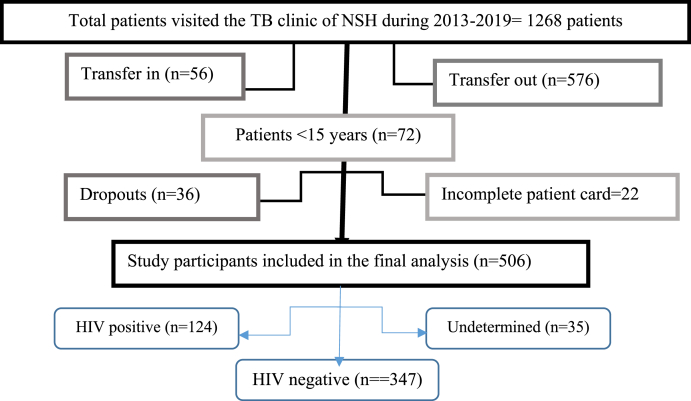
Flow diagram of the eligible TB patients included in the final analysis, 2019.

In NSH, over 6 years of the study period, a total of 1268 TB patients were enrolled for treatment. From these patients, 56 transfer in, 576 transfer out, 36 drops out, 72 children less than 15 years, and 22 incomplete patient cards were excluded from the study resulting in 506 eligible patients. The samples of 506 patient registration cards were selected by a convenient method (Fig. 1).

### 2.7 Data collection process

A semi-structured data collection format that contains sociodemographic characteristics, disease-related factors, medication-related factors, and treatment outcome was prepared to extract the data from patients' medical records. The questionnaire contains socio-demographic factors, type of tuberculosis infection, history of exposure to anti-tuberculosis treatment, the status of HIV infection, and the outcome of anti-tuberculosis treatment. Information on ART CD4, HIV care, HIV stage, and provision of CPT for HIV-infected TB patients were recorded in the ART register. The socio-demographic data, HIV status, TB enrollment, category of TB at the start, and the treatment outcomes of the TB patients were collected from the TB Unit registers. Information on the primary endpoint (treatment success) was based on information written on patient's cards and review of the document if there were death certificates. The patients' date of death was extracted from TB registration logbooks. Patients were followed from since the start of anti-TB medications lost to follow-up, treatment completion and died. Data were collected by two nurses working at the TB clinic after being trained about the objectives of the study, data collection format, and techniques of data extraction.

### 2.8 Data quality control and management

To ensure the data quality, the trained data collectors were informed on eligibility criteria of the study participants, and recording of the right information from the hospital registration book and/or patient's follow-up medical chart to maintain the completeness, accuracy, and consistency of the collected data. Data abstraction checklist was pre-tested at Wollega University referral hospital which provides similar services to the TB patients before the commencement of the actual data collection. Completeness of the data was checked, coded, and entered into the computer using SPSS version 24 statistical software daily. Mode, median and mean values were used to address incompleteness, inconsistencies, and inaccuracy depending on the measurement scale. Each entry was cross-checked independently to ensure the quality of data. Supervisors, data clerks, and investigators had checked completeness and consistency of data before and after data entry.

### 2.9 Data processing and analysis

The data was entered into the computer using Epidata version 3.1 and analysis was done using statistical package for social sciences (SPSS) 24. Descriptive statistics (frequency, proportion, mean and standard deviation) were used to summarize patients’ characteristics of the cohort. Multivariate analysis using a logistic regression model was used to analyze the association between treatment outcome and potential predictor variables. All variables were entered into multivariable logistic regression (LR) using a backward LR method to control the confounding effect. Explanatory variables that had a significant association with the outcome at p-value less than 0.25 in the bi-variable binary logistic regression were entered into the multivariable logistic regression model. Association between outcome and predictor variables was calculated using adjusted odds ratio and the statistical significance was considered at p < 0.05 for associated variables within a 95% confidence interval. Assumption of the goodness of the model was checked by Hosmer Lemeshow test (p = 0.631).

### 2.10 Ethical approval and consent to participate

Ethical approval was obtained from the Research Ethics Review Committee (RERC) of the Institute of Health Sciences of Wollega University with reference number: 023CHRT/11. A full waiver of informed consent was granted since patients were not encountered directly in the study. Data were extracted from patient treatment folders stored in the hospitals. Staff members of the card room of NSH permission for any cooperation will be politely asked. The confidentiality of patients is secured throughout the study periods using code instead of their name. Institutional ethics and an individual's privacy of the patient were respected. Finally, the questionnaires were kept locked after data entry was completed. The Unique Identifying Number (UIN) of this study is researchregistry6188 in Research Registry registration, in accordance with the declaration of Helsinki [51].

### 2.11 Operational definitions

According to The Ethiopian National TB Control Program (NTLP) guideline adopted from WHO, the following standard clinical case definition and treatment outcome rate operational terms were used [1,10,18,46,52].

Cases of TB.

• **Smear-positive pulmonary TB** (PTB+)**:** A patient with at least two sputum specimens which were positive for acid-fast bacilli (AFB) by direct microscopy, or a patient with only one sputum specimen which was positive for AFB by microscopy, and chest radiographic abnormalities consistent with active pulmonary TB as determined by a clinician.
• **Smear-negative pulmonary TB (PTB-):** A patient with symptoms suggestive of TB, with at least three sputum specimens negative for AFB by microscopy, and with chest radiographic abnormalities consistent with active pulmonary TB, and lack of clinical response to one week of broad-spectrum antibiotic therapy, or a patient whose diagnosis is based on culture-positive Tuberculosis but with three initial smear examinations negative by direct microscopy.
• **Extra-pulmonary TB (EPTB)**: This included tuberculosis of organs other than the lungs, such as lymph nodes, abdomen, genitourinary tract, skin, joints and bones, meninges, etc. Diagnosis of EPTB was based on fine-needle aspiration cytology or biochemical analyses of cerebrospinal/pleural/ascitic fluid or histopathological examination or strong clinical evidence consistent with active extra-pulmonary tuberculosis, followed by a decision of a clinician to treat with a full course of anti-tuberculosis chemotherapy. A patient in whom both pulmonary and extra-pulmonary TB has been diagnosed should be classified as a pulmonary case.

TB treatment outcome.

• **Cured:** A patient who was initially sputum smear-positive and who was sputum smear-negative in the last month of treatment and on at least one previous occasion;
• **Treatment completed:** A TB patient who completed treatment without evidence of failure or cure. Patients with documented treatment completion and resolution of symptoms, but for whom sputum smear, culture results are not available in the last month of treatment and on at least one previous occasion.
• **Died:** when a patient died from any cause during treatment.
• **Treatment failure**: when a patient was initially sputum smear-positive and when a patient remained sputum smear-positive at for 5 months or later during treatment
• **Transferred out**: A patient whose treatment outcome is unknown due to transfer out to another health facility.
• **Defaulted (**interrupted patient**)**: A patient who has been on treatment for at least 4 weeks and whose treatment was interrupted for two consecutive months or more for any reason without medical approval.
• **Relapse:** It is a patient who has been declared cured or has completed treatment of any form of TB in the past but who reports back and was found to be smear-positive.
• **Successful treatment outcome (treatment success):** It is the sum of the patients who are cured or completed treatment.
• **Unsuccessful/unfavorable/poor outcome:** The sum of patients who are defaulter, died and patients with treatment failure

***Functional status [***17***]:*** was measured at base-line as:

• **Working:** Able to perform usual work in or out of the house
• **Ambulatory:** Able to perform activities of daily living
• **Bedridden**: Not able to perform activities of daily living.

## 3 Results

### 3.1 Socio-demographic characteristics of study participants

A total of 1268 TB patients were registered at NSH from 2013 to 2019 years. A total of 506 TB patients, including 357 non-HIV infected, 124 HIV co-infected, and 35 patients with undetermined HIV status fulfilling eligibility criteria were enrolled in the final analysis. Out of the total study participants, about half (50.2%) of them were males and the mean age of the study participants was 33 ± 12.4 years. About one third (36%) of them were in the modal age range of 25–34 years. About two-thirds (66%) of the study participants were married and 30% had college and above educational level (Table 1).

**Table 1:** Socio-demographic characteristics of the study participants followed at Nekemte specialized hospital, 2013–2019.

Sociodemographic characteristics		Frequency(n)	Percentage (%)
Sex	Male	254	50.2
	Female	252	49.8
Age (years)	15–24	128	25.3
	25–34	182	36
	35–49	122	24.1
	≥50	74	14.6
Marital Status	Married	334	66
	Single	139	27.5
	Others (widowed, divorced)	33	6.5
Religion	Protestant	250	49.4
	Orthodox	230	45.5
	Muslim	24	4.7
	Catholic	2	0.4
Educational Status	No formal education	134	26.5
	Elementary	84	16.6
	High school	136	26.9
	College and above	152	30
Employment Status	Employed	146	28.9
	Self-employed	143	28.3
	House wives	98	19.4
	Unemployed	94	18.6
	Farmers	25	4.9
Residence	Urban	456	90.1
	Rural	50	9.9

### 3.2 Clinical and medication characteristics of the study participants

Among TB patients at the start of treatment, 88.1% were new cases, 8.3% were relapsed, 2.0% were failed and 1.6% were returnees after default. Overall, there were 60 (11.9%) retreatment cases. Based on the type of TB, 23.9% of the patients were diagnosed as EPTB, 38.1% smear-negative PTB, and 37.9% smear-positive PTB.

Regarding the HIV related characteristics, about one fourth (24.5%) of the study participants were HIV positive. From HIV positive participants, the majority (84.7%) of them were on ART during their TB course of treatment. About three-fifth (58.9%) of the study participants were in WHO stage IV. Prophylactic treatment with co-trimoxazole is part of the standard of care for HIV co-infected patients receiving TB treatment and given for 110 (88.7%) patients (Table 2).

**Table 2:** **C**linical and medication characteristics of the tuberculosis patients at Nekemte specilaized hospital, 2013–2019.

HIV related characteristics		Frequency(n)	Percentage (%)
Type of TB	PTB+	192	37.9
	PTB-	193	38.1
	EPTB	121	23.9
TB registration status (TB enrollment)	New	446	88.1
	Relapse	42	8.3
	Treatment failure	10	2.0
	Treatment after default	8	1.6
INH Propylaxis	Yes	33	6.5
	No	324	93.5
HIV status	Reactive	124	24.5
	Non-reactive	347	68.6
	Indeterminate	35	6.9
CD4 count during TB diagnosis (Cells/mm^3^) [n = 124]	≥200	21	17
	50–199	37	30
	<50	66	53
WHO HVI stage during TB diagnosis [n = 124]	Stage I	6	4.9
	Stage II	9	7
	Stage III	36	29
	Stage IV	73	58.9
Enrolled in HIV care [n = 124]	Yes	115	92.7
	No	9	7.3
CPT given [n = 124]	Yes	110	88.7
	No	14	11.3
Started ART [n = 124]	Yes	105	84.7
	No	19	15.3
ART regimen at TB diagnosis [*n* = 124]	TDF-3TC-NVP	65	52.42
	TDF-3TC-EFV	37	29.84
	D4t-3TC-NVP	9	7.26
	AZT-3TC-EFV	5	4.03
	ABC-3TC-EFV	5	4.03
	AZT-3TC-NVP	3	2.42
	Second line regimens	65	52.42
INH Propylaxis	Yes	64	12.6
	No	442	87.4
Functional status	Working	341	67.4
	Ambulatory	125	24.7
	Bedriden	40	7.9
Nutritional status	Malnourished	191	37.7
	Not malnourished	315	62.3
Presence of comorbidity (n = 232)	HIV	124	53.4
	Other RTI (including Asthma)	31	13.3
	Cardiovascular disorders (HTN, IHD, HF)	27	10.3
	Diabetes Mellitus	21	9.1
	Pschiatric disorders (depression and anxiety	16	6.9
	Renal diseases	9	3.9
	Anemia	4	1.7
Treatment associated adverse effects (toxicity)	yes	yes	143
	No	No	363

ART: Antiretroviral treatment; CD4: Cluster differentiation-4, HIV: Human immune virus; WHO: World health organization. EPTB: Extra-pulmonary Tuberculosis; TB: Tuberculosis; PTB+: Pulmonary positive TB, PTB-: Pulmonary negative TB; INH: Isoniazid; CPT: Cotrimoxazole preventive therapy, ABC: Abacavir; TDF: Tenofovir fumarate; 3 TC: Lamivudine, D4T: Stavudine; AZT: Zidovudine; EFV: Efavirenz; NVP: Nevirapine; HTN: Hypertension; IHD: Ischemic heart diseases; HF: Heart failure; RTI: Respiratory tract infection.

### 3.3 Treatment outcomes of TB patients

The rate of cure among all forms of TB cases was 72 (14.2%) while the rate of treatment completion was 340 (67.2%). The rate of treatment failure, default, and death was 32(6.3%), 20(4%), and 42 (8.3%), respectively. The cure rate was significantly lower (6.4% versus 17.6%) and the death rate was higher in HIV co-infected patients (19.4% versus 2.9%) which was statistically significant (p < 0.001) (Table 3).

**Table 3:** Treatment outcomes of TB patients enrolled at Nekemte specialized hospital, 2013–2019.

HIV status	TB treatment Outcome					
	Cure	Treatment Completed	Defaulted	Treatment failure	Death	*p*-value
HIV negative, n (%)	61 (17.6)	254 (73.2)	11 (3.2)	11 (3.2)	10 (2.9%)	0.000
HIV positive, n (%)	8 (6.4)	65 (52.4)	9 (7.3)	18 (14.5)	24 (19.4)	

**Fig. 2:**
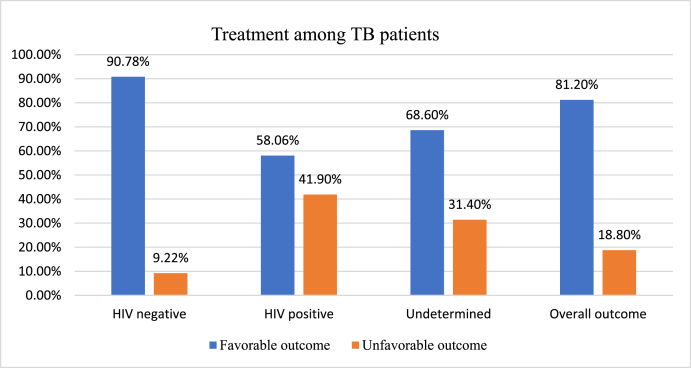
Comparison of TB treatment success based on HIV status at NSH, 2013–2019.

Analysis of the study data demonstrated that the overall TB treatment success rate was 81.4%. This finding persists at 82.16% treatment success rate even after excluding 35 those with undetermined HIV status. However, the treatment success rate among HIV co-infected TB patients was 58.06% (Fig. 2).

### 3.4 Impact of HIV status and factors affecting TB treatment outcome

Both bivariable and multivariable analysis was done to identify the association between independent variables and TB treatment outcome. On multivariable logistic regression: sex, age, marital status, educational level, residence, TB category, TB enrollment, and HIV status were significantly associated with successful TB treatment outcomes.

The likelihood of successful TB treatment outcome was nearly four times (AOR = 3.992, 95%CI; 1.743–9.145, p = 0.001) more likely among female patients compared to males. The treatment of TB among patients whose age between 25 and 34 years were about 4 times (AOR = 3.982, 95% CI: 1.445–10.971, p = 0.008) and age 35–49 were about five times (AOR = 5.392, CI: 1.674–17.4, p = 0.005) more likely to be successful as compared to patients whose age was ≥50 years. The odds of successful TB treatment was significantly low among single participants (AOR = 0.293, 95CI: 0.1–0.854, p = 0.025) as compared to married ones. The educational status of the patients was also another factor that was associated with TB treatment outcomes. Higher educational status like college and above were nearly four times (AOR = 3.751, 95% CI, 1.011–13.922) more likely to be successful. Patients were more likely to have a successful treatment if they were urban residents (AOR = 3.093, 95%, 1.003–9.541, p = 0.049) compared to rural residents. Successful treatment outcome was less frequent among smear-negative patients (AOR = 0.360, 95% CI: 0.156–0.834, p = 0.017) and EPTB (AOR = 0.839, 95% CI: 0.560–0.955, p = 0.031) than smear-positive pulmonary TB patients. Patients were less likely to have a successful treatment if they were retreatment cases (AOR: 0.54, 95% CI: 0.004–0.098, p < 0.001) compared to patients with new TB cases. HIV negative patients were about 10 times more likely to experience treatment success (AOR = 10.3, 95% CI, 3.216–32.968, p < 0.001) compared to HIV positive TB patients (Table 4).

**Table 4:** Factors affecting successful TB treatment outcome and its association with HIV status at NSH, 2013–2019.

Variable		TB treatment outcome		COR (95% CI)	AOR (95% CI)	*P*-Value
		Successful	Not successful			
Sex	Male	190	64	1	1	
	Female	221	31	2.4 (1.5–3.85)	3.992 (1.743–9.145)	.001*
Age (years)	15–24	109	19	4.136 (2.11–8.09)	2.702 (.808–9.038)	.107
	25–34	158	24	4.746 (2.53–8.92)	3.982 (1.445–10.971)	.008*
	35–49	101	21	3.46 (1.79–6.79)	5.392 (1.674–17.368)	.005*
	≥50	43	31	1	1	
Marital Status	Married	276	58	1	1	
	Single	107	32	0.70 (0.43–1.14)	.293 (.100–.854)	.025
	Others (widowed, divorced)	28	5	1.18 (0.44–3.18)	3.483 (.649–18.698)	.146
Educational level	No formal education	131	15	1	1	
	Elementary	104	39	3.95 (1.92–8.15)	3.246 (.986–10.688)	.053
	High school	20	5	4.80 (2.53–9.11)	5.330 (1.753–16.209)	.003*
	College and above	81	17	4.17 (2.30–7.55)	3.751 (1.011–13.922)	.048*
Occupational Status	Employed	131	15	1	1	
	Self-employed	104	39	0.31 (0.16–0.58)	.864 (.227–3.290)	.831
	Farmers	20	5	0.46 (0.15–1.40)	1.070 (.121–9.472)	.952
	House wives	81	17	0.55 (0.26–1.15)	1.120 (.289–4.337)	.869
	Unemployed	75	19	0.45 (0.22–0.94)	.219 (.058–1)	.051
Residence	Urban	378	78	2.5 (1.32–4.7)	3.093 (1.003–9.541)	.049*
	Rural	33	17	1	1	
Nutritional status	Malnourished	124	67	1	1	
	Not malnourished	287	28	0.18 (0.11–0 .29)	9.514 (3.760–24.075)	.085
TB class	PTB+	151	41	1	1	.010
	PTB-	150	43	0.95 (0.58–1.54)	.360 (.156–.834)	.017*
	EPTB	110	11	2.72 (1.34–5.52)	0.839 (.560–.955)	.031*
TB enrolment	New	362	84	1		
	Retreatment	44	16	.64 (0.01–0.09)	.54 (.004–.098)	.000*
Comorbidity	Yes	166	66	1	1	
	No	245	29	3.36 (2.08–5.42)	1.537 (.389–6.070)	.539
HIV status	HIV+	72	52	1	1	
	HIV-	315	32	7.11 (4.27–11.8)	10.3 (3.216–32.968)	.000*
	Not determined	24	11	1.58 (0.71–3.50)	2.258 (.631–8.076)	.210

AOR: Adjusted odds ratio; COR: Crudes odds ratio; HIV: Human immune virus; EPTB: Extra-pulmonary Tuberculosis; TB: Tuberculosis; PTB+: Pulmonary positive TB, PTB-: Pulmonary negative.

*Statistically significant at p < 0.05.

## 4 Discussion

TB becomes a major public health problem throughout the world particularly in developing countries [27,36]. The presence of HIV infection increases the chance of tuberculosis reactivation and infection due to TB [22]. In this study, the magnitude of HIV was 24.5% which is similar to the finding of Harar town 22.8% [27], Felege Hiwot referral hospital (25%) [5], and Debre Tabor general hospital (24.2%) [48]. However, the prevalence was higher than previous studies at rural Ethiopian hospitals (1.7%) [22], Aurangabad city (7.28%) [26], and WHO 2019 TB report (8.6%) [7]. This high TB-HIV coinfection could be due to the higher proportion of patients from urban settings where there is a high prevalence of HIV. Additionally, the proportion of TB-HIV coinfection was much lower than the previous report from Gondar (52.1%) [53] due to differences in study design and socioeconomic characteristics of study participants. In our current study, only 23.9% were presented with EPTB and this finding was contrary to previous studies where the majority of the patients where EPTB patients [5,27]. This low proportion of EPTB in our study might be due to less diagnosis or misclassification bias.

In the current study, the proportion of TB patients with successful treatment outcomes was found to be 81.2% which was comparable with studies reported from Addis Ababa (82.7%) [54], Nekemte Referral Hospital (82.5%) [45], Sodo town (81.5%) [8], Eastern Ethiopia (81.8%) [1], Central Ethiopia (83.6%) [55], New Delhi, India (81%) [16], Abeokuta (83.5%) [41], Lubumbashi DR Congo (83.3%) [3], international epidemiology databases (80%) [29] and a systematic review and meta-analysis by Eshetie et al. (83.7%) [20]. This overall rate of the treatment success was lower than studies conducted by Mekonnen et al. (86.2%) [25], Gebremariam et al. (87.3%) [6], Ejeta et al. (85.2%) [10], Tola et al. (92.5%) [27], Endris et al. (94.8%) [4], Melese et al. (87.1%) [56], Ali et al. (91.5%) [44], Beza et al. (85%) [57], Worku et al. (90.1%) [48], Abebe et al. (88.2%) [58] and Tweya et al. in Malawi (86%) [59]. Additionally, in this study, the successful treatment outcomes was lower than NTLCP and WHO target set for the Millennium Development Goal (MDG) of 85% [2,60,61] and far lower to that of ≥90% End TB Strategy success rate of milestone target set globally for 2025 [62,63]. The possible explanation for this discrepancy might be associated with the difference in study design, characteristics, and the number of study participants involved in the study. On top of that, the handling of transfer out cases as one of the case definitions of the TB treatment outcome in earlier various studies might affect the successful treatment outcome. Besides, the substantial figures of death recorded, and socio-economic conditions of the society in our cases may contribute to the low treatment outcome.

However, the rate of successful treatment outcome was higher than studies conducted by Biadglegne et al. (26%) [5], Shargie and Lindtjørn (49.5%) [64], Tessema et al. 29.5% [30], Rocha et al. (77.1%) [22], Fiseha et al. (28.9%) [14], Asebe et al. (70.76%) [32], Ejeta et al. (70.8%) [47], Kefale et al. (30.32%) [15], Warkari et al. (63.21%) [26], Adebimpe et al. (78.7%) [23] and Ansa et al. (64.0%) [65]. The satisfactory treatment success rate in our study might be due to improved adherence of TB patients associated with a good implementation of DOTs strategy, which was indicated by relatively lower failures and default rates. One explanation for a high success rate in the current study might due to exclusion of transfer out cases unlike the previous study [56], which included in the analysis. Additionally, the majority of the TB patients in this study were urban residents who can easily access the health facilities to follow their treatment and had better health-seeking behavior as compared to rural.

The treatment success rate among HIV co-infected TB patients was 58.06%, which was comparable with findings at western Ethiopia (60.7%) [12], South-eastern Nigeria (64.1%) [11] and India 66% [66], but higher as compared to study at Mizan-Aman general hospital (28.9%) [14] and Felege Hiwot Referral Hospital (22.5%) [5]. Additionally, the treatment success rate among TB-HIV co-infected patients was lower compared to study at Harar (86.8%) [38], Addis Ababa 88.2% [44], Debre Tabor 88.1% [48] and Gondar (77.3%) [18]. WHO global TB report of 2011 indicated in 2009 the TB treatment success rate of 72% and 88% for HIV co-infected and non-HIV infected TB patients, respectively [67]. But, according to the global TB report of 2012, HIV co-infected TB patients had 74% favorable treatment outcomes compared with 88% for HIV-negative TB patients [68]. Additionally, according to WHO 2017 Global Tuberculosis report, the global treatment success rate for HIV-associated TB cases among the 2015 cohort was 78% and in the WHO Africa region, it was 80% [69]. The low treatment success rate observed in the present study might be due to the high treatment default rate, failure, and death rate among the co-infected patients. The difference between our findings and results of others might be due to the presence of TB and HIV drug-drug interaction, the patient's knowledge on the necessity of adhering to TB treatment, and the presence of facilities used to diagnose and treat these comorbidities [18].

TB associated death rate in the present study was 8.3% which was in line with the report by Tola et al. (7.7%) [36], Beyene et al. (9.3%) [19], and Ejeta et al. (8.1%) [47]. On the contrary, the death rate was lower as compared to the study conducted by Kefale et al. (12.77%) [15], Tessema et al. (10.1%) [30], and Akanbi et al. (10.1%) [41]. Additionally, some studies reported lower proportion of TB associated death rate, ranging from 3.6% in Arsi Negele Health Center to 6.3% in West Ethiopia [1,5,8,14,27,32,59]. Among those died, 17.6% were HIV positive patients. Similar findings were reported from Dessie Referral Hospital (18%) [19] and Felege Hiwot Referral Hospital (15.2%) [5]. However the finding was higher compared to study at eight health facilities in Addis Ababa (8.3%) [44]. Poor management of the health care system, non-adherence to their treatments, and shortage of DOTS could have contributed to unfavorable treatment outcomes. Overall, the difference in the mortality rates at different setups might be due to institutional factors, patient-related factors, and methods of study employed. Hence, strengthening monitoring, supervision, and health education to reduce deaths should be among the top priorities in the study area.

The other main challenge of the TB controlling program is treatment failure. In the current study, 6.3% of the TB patients were identified as treatment failure [27]. This figure was comparable with study at West Ethiopia (7.6%) [45] and lower as compared to study at South-western Nigeria (12.8%) [23]. Unlike our findings, other previous studies revealed lower rates of treatment failure ranged between 0.2% in Western Ethiopia to 3.7% at public hospitals of Harar town [1,5,6,8,27,30,32,36,41,47,59]. The higher treatment failure in our study area might be related to variables like the severity of the disease, timing of the treatment, adherence to the treatment, available facilities, recording of patients’ history and behavioral as well as socioeconomic characteristics of study participants.

Defaulting from the anti-TB treatment clinic is one of the other challenges of the TB controlling program. The default rate of TB patients in the current study was 4%, which is comparable with the findings from Abeokuta, Nigeria (4.6%) [41] and West Ethiopia (3.5%) [45]. The figure was higher than reports from Felege Hiwot Referral Hospital (2.5%) [5], Arsi Negele health center (1.7%) [6], and Mizan-Aman general hospital (1.3%) [14]. However, the majority of previous studies reported that higher defaulting rate ranged from 5.9% in Sodo town (32) to 18.3% in Gondar University Teaching Hospital [1,8,12,15,30,32,47]. This lower defaulter rate in this study might be due to proper supervision and better health education.

Understanding and identification of factors associated with treatment outcome of TB patients are very indispensable to overcome those factors leading to poor treatment outcomes and can help to design an appropriate evidence-based intervention to reduce morbidity and mortality [27]. Upon multivariate regression analysis, the treatment outcome was varied with sex, age, marital status, educational level, residence, Tb category, TB enrollment, and HIV status.

Age was found to be one predictor factor for successful treatment outcomes, where the age group of 25–49years was associated with favorable outcomes. Previous studies also supported that being an older age (increased age) was associated with a lower treatment success rate including a higher risk for mortality that needs to be addressed urgently [1, 5, 10, 30, 35, 44, 70, 71]. Global TB 2012 reported that TB patients ≥65 years of age had a higher likelihood of unfavorable outcome than those in the other age groups [44,72]. Older age has been reported to be a risk factor for death, partly due to comorbidities as well as the general physiological deterioration with increasing age [30,31],. However, other studies revealed that odds of successful treatment outcome was higher among older patients compared to younger patients [9,11,30,56] which might be because TB/HIV co-infection affects the reproductive age group.

In the present study, female patients were more likely to have successful treatment outcomes compared to male patients and this finding was in line with many previous studies [1,27,30,73,74]. The higher social interaction outside the home by males, social isolation associated with treatment rejection, use of social drugs, and other related behaviors among males might contribute to their higher default, failure, and death. Additionally, most females stay at home and need their drugs compared to males who are always busy with playing with their peers. Unlike the current finding, a study by Beyene et al. showed about two-fold risk of mortality among female TB patients compared to males [19]. This difference might be related to socio-economic and socio-cultural factors affecting women mostly in developing countries such as poor quality of life, overcrowding, under-nutrition, low level of education and empowerment as well as social placement of women as inferior to men.

The odd of successful TB treatment was significantly low among patients who were single as compared to those who were married. This was similar to a study conducted in Somali and Turkey where married patients had higher successful treatment [75,76]. This could be because patients lack family support and therefore miss their medication.

The educational status of the patients was also another factor that was associated with TB treatment outcome and higher educational status patients were more likely to be successful. This corresponds to findings conducted in Somalia and Turkey where patients with low education had higher unfavorable treatment outcome [75,76]. The differences between educated and less-educated might be due to the difference in living styles, knowledge level, economic background, communication strategies, and clinical condition. Education is perceived to reduce ignorance, increase knowledge on drugs management, transmission route, preventive measures, and suspicious symptoms that results in good medication compliance. Low education level is also associated with poor health, low self-management behaviors, and lower continuity of care.

In this study, the patients from urban areas had a significantly higher treatment success rate compared to rural residents which was in line with studies reported elsewhere [5,10,18,30,41,56,77]. The lower treatment success rate in rural patients could be due to lower awareness of TB treatment, poor socioeconomic status, and the long-distance from the treatment center due to the unavailability of nearby health institutions. Additionally, fear of stigma and discrimination outside of the town might be contributed to unfavorable TB treatment outcomes.

The present study demonstrated that there were higher odds of unsuccessful treatment outcome among EPTB and PTB- compared with PTB + which was consistent with the results of other studies [5,6,10,15,56,58,78]. The finding might be attributed to delayed diagnosis and initiation of treatment in PTB- and EPTB patients, where patients' treatment response was managed only clinically [79]. In PTB+, sputum tests have immense contribution to know the patients’ treatment response on time and manage the condition to prevent further morbidity and development of drug resistance The existing literatures also showed a higher prevalence of HIV in patients with PTB- and EPTB [6,80,81]. However, other studies revealed that PTB + patients had higher unsuccessful treatment outcomes than EPTB and PTB- [8,36,44,82]. The relatively lower overall successful treatment among smear-positive PTB in these studies could be attributed to poor smear microscopy resulting in false PTB- and lack of tracing out of the treatment outcomes of defaulted patients.

The study also revealed that the risk of unsuccessful TB treatment outcome was significantly higher among retreated cases compared to newly registered and the finding was in agreement with previous studies [10,20,27,36,41,75,76]. Patients with previously treated cases have a high level of treatment failure as a result of possible development of drug resistance in the repeated exposure [10]. This could be due to due to improper use of drugs by TB patients associated with administration of wrong treatment regimens and poor adherence to anti-TB drugs.

The HIV status of TB patients was one of the factors that was associated with the treatment outcome. TB/HIV co-infection was significantly associated with poor TB treatment outcome in the present study and this finding was consistent with several previous studies [1,6,11,19,20,25,27,34,35,78,83]. Several possible explanations could be proposed for the striking difference in treatment success between patients with TB only and those co-infected with HIV. Concomitant administration of anti-TB and ART drugs, which can lead to default from higher pill burden, poor patient compliance, and missing pills due to fear of side-effects [9,18]. All of these factors can lead to treatment failure, drug interactions, overlapping toxic effects, immune reconstitution syndrome and ultimately death [9,31]. The presence of other morbidities like neoplastic diseases and other opportunistic diseases in HIV positive TB patients also implicated in the increased mortality [84]. Immunological studies have also shown that the host responses to Mycobacterium tuberculosis enhance HIV replication. Thus, accelerating the natural progression of HIV and further decreasing cellular immunity [19,85].

HIV infection is one of the major reasons for the unfavorable tuberculosis treatment success rate (at least 85% cure rate among new sputum SPTB + cases) in countries with a high burden of HIV infection [19]. This is attributable to factors such as overdiagnosis of sputum SNPTB, under-diagnosis of SPPTB low cure rates, high mortality and default rates during treatment as well as atypical clinical presentation of TB in HIV infected patients. While this is true, our results suggest that the implementation of program guidelines in a coordinated manner can result in good treatment outcomes among those co-infected with TB and HIV. Hence, early diagnosis and prompt treatment among people living with HIV is paramount for decreasing TB associated morbidity and mortality.

### 4.1 Limitation of the study

Our study was subject to several important limitations. First, this study was related to the use of retrospective secondary data which in some way proved to have some issues with missing and getting inaccurate data. Some important variables which might have an impact on TB treatment outcome like socioeconomic characteristics (weight, height, income, family size, living condition, social support, distance to the health facility), treatment and disease-related variables (adherence level, viral load, drug resistance, presence of other comorbidities, specific types of EPTB, the severity of immune suppression), as well as behavioral factors (knowledge and attitude about the diseases, alcohol abuse, cigarette smoking, illicit drug use) were not recorded.

Second, we evaluated patients treated at a single institution, not reflecting approaches at other medical centers that require caution in generalizing for a large community. Hence, a prospective and multi-centric study is required to validate our results. Third, the data have been extracted from medical records of patients who have been already visited and registered at the hospital, so it may be subjected to selection bias. Additionally, TB-HIV co-infected patients who started ART before initiating TB treatment, and those who started ART while being treated for TB, were included in the same group which may also introduce bias. Finally, the exclusion of medical records of a patient who were transferred out and/or found to be incomplete may have also slightly affected our results.

## 5 Conclusion

In summary, the treatment success rate of TB patients treated at NSH was 81.2%, lower than NTLCP, and WHO target set for the Millennium Development Goal of 85%. Furthermore, the odds of successful treatment outcome were higher among patients 25–49 years of age, females, married, urban residents, patients with better education level, HIV negative, PTB + and new cases as compared to their counterparts. The findings of this study also demonstrated statistically significant differences between the treatment outcome rates among TB/HIV co-infected patients and TB-HIV negatives patients.

The increased unsuccessful treatment outcome among TB/HIV patients requires urgent public health interventions and the strengthening of existing control programs to improve the evaluation policy and control framework among TB and HIV co-infected patients. This integrated and coordinated TB control program that includes active case surveillance, effective care and treatment, and quality laboratory diagnosis services reduces morbidity and mortality. Optimal provision of quality care, early identification, and prompt initiation of treatment for both TB treatment and HIV patients will help in bringing down the morbidity and mortality associated with TB-HIV co-infection. Hence, interventions should also direct at modifiable risks such as treatment of opportunistic infections and other concomitant illnesses or conditions.

Frequent supportive supervision and health education should be given for the society on the mode of transmission of TB, prevention of infection, benefits medication adherence, the impact of TB-HIV co-infection on TB treatment outcomes. Additionally, actions targeting predictor factors like education for rural residents and good coverage of diagnostic agents as well as the quality of laboratory services for EPTB are necessary. The data registration system of patients should be modified to include patients’ practices like smoking habits, alcoholism and other chronic illness. Furthermore, future prospective and qualitative studies are needed to identify other potential sociodemographic, medication, clinical, delivery of services, patient compliance, and behavioral factors that could affect the treatment outcomes of TB patients.

Since our study was conducted in a single setup, collaborative projects that can incorporate broad areas with large samples are recommended to give a more balanced view of factors influencing TB treatment outcomes and impacts of HIV on treatment.

## Ethical approval

Ethical approval was obtained from the Research Ethics Review Committee (RERC) of the Institute of Health Sciences of Wollega University with reference number: 023CHRT/11. A full waiver of informed consent was granted since patients were not encountered directly in the study. Data were extracted from patient treatment folders stored in the hospitals. Staff members of the card room of NSH permission for any cooperation will be politely asked. The confidentiality of patients is secured throughout the study periods using code instead of their name. Institutional ethics and an individual’s privacy of the patient were respected. Finally, the questionnaires were kept locked after data entry was completed.

## Funding

This research did not receive any specific grant from funding agencies in the public, commercial, or not-for-profit sectors.

## Author contribution

GF* was the primary researcher, conceived the study, designed, participated in data collection, conducted data analysis, and drafted the manuscript for publication. ET, TK, FB, LC and TT assisted in data collection, supervision, analysis and preparation of the first draft of the manuscript. BG, DD, GF and DCD validated, drafted, interpreted the results, and reviewed the initial and final drafts of the manuscript. All authors critically revised the manuscript and have approved the final manuscript.

## Registration of research studies

Ginenus Fekadu. Registration of research studies.

1. Name of the registry: RESEARCH REGISTRY, https://www.researchregistry.com
2. Unique Identifying number or registration ID: researchregistry6188
3. Hyperlink to your specific registration (must be publicly accessible and will be checked):https://www.researchregistry.com/browse-the-registry#home/registrationdetails/5f9d14da5e2b290015631061/

## Guarantor

Ginenus Fekadu

## Consent for publication

Not applicable. No individual personal details, images or videos are being used in this study.

## Provenance and peer review

Not commissioned, externally peer reviewed.

## Availability of data and materials

The datasets used and/or analyzed during the current study are available from the corresponding author on reasonable request.

## Declaration of competing interest

The authors declare that they have no competing interests.

## Abbreviations

AFB: Acid-fast bacilli
ART: Antiretroviral treatment
CD4: Cluster differentiation-4
CPT: cotrimoxazole prophylactic therapy
DOTs: directly observed therapy
EPTB: Extra-pulmonary Tuberculosis
HIV: Immunodeficiency Virus
LMICs: Low and middle-income countries
NSH: Nekemte Specialized Hospital
NTBLCP: National Tuberculosis and Leprosy Control Programme
PTB: Smear-negative pulmonary TB
PTB+: Smear-positive pulmonary positive TB
SSA: Sub Saharan Africa
TB: Tuberculosis
TB: Tuberculosis
WHO: World health organization

## References

[1] Zenebe T., Tefera E. (2016). Tuberculosis treatment outcome and associated factors among smear-positive pulmonary tuberculosis patients in Afar, Eastern Ethiopia: a retrospective study. Braz. J. Infect. Dis..

[2] World Health Organization (2016). Global Tuberculosis Report. https://apps.who.int/iris/handle/10665/250441.

[3] Kakisingi C., Mukuku O., Kajimb P. (2018). Treatment outcome of Tuberculosis patients with HIV under directly observed treatment short course (DOTS) in Lubumbashi (DR Congo). J Infectious Disease Med Microbiol.

[4] Endris M., Moges F., Belyhun Y., Woldehana E., Esmael A., Unakal C. (2014). Treatment outcome of tuberculosis patients at Enfraz Health Center, Northwest Ethiopia: a five-year retrospective study.

[5] Biadglegne F., Anagaw B., Debebe T., Anagaw B., Tesfaye W., Tessema B., Rodloff A.C., Sack U. (2013). A retrospective study on the outcomes of tuberculosis treatment in Felege Hiwot Referral Hospital, Northwest Ethiopia. Int. J. Med. Med. Sci..

[6] Gebremariam G., Asmamaw G., Hussen M., Hailemariam M.Z., Asegu D., Astatkie A., Amsalu A.G. (2016). Impact of HIV status on treatment outcome of tuberculosis patients registered at Arsi Negele health center, southern Ethiopia: a six year retrospective study. PLoS One.

[7] World Health Organization (2019). Global Tuberculosis Report 2019. https://apps.who.int/iris/bitstream/handle/10665/329368/9789241565714-eng.pdf?ua=1.

[8] Yakob B., Alemseged F., Paulos W., Badacho A. (2018). Trends in treatment success rate and associated factors among tuberculosis patients in Ethiopia: a retrospective cohort study. Health Sci. J..

[9] Azeez A., Ndege J., Mutambayi R. (2018). Associated factors with unsuccessful tuberculosis treatment outcomes among tuberculosis/HIV coinfected patients with drug-resistant tuberculosis. International Journal of Mycobacteriology.

[10] Ejeta E., Beyene G., Balay G., Bonsa Z., Abebe G. (2018). Factors associated with unsuccessful treatment outcome in tuberculosis patients among refugees and their surrounding communities in Gambella Regional State, Ethiopia. PLoS One.

[11] Babatunde O.I., Christiandolus E.O., Bismarck E.C., Emmanuel O.I., Chike A.C., Gabriel E.I. (2016). Five years retrospective cohort analysis of treatment outcomes of TB-HIV patients at a PEPFAR/DOTS Centre in South Eastern Nigeria. Afr. Health Sci..

[12] Ejeta E., Birhanu T., Wolde T. (2014). Tuberculosis treatment outcomes among tuberculosis/human immunodeficiency co-infected cases treated under directly observed treatment of short course in Western Ethiopia. J. AIDS HIV Res..

[13] Amare D. (2015). Tuberculosis and HIV Co-infection among patients on tuberculosis treatment at fenote selam district hospital, Amhara regional state, northwest Ethiopia. Global J. Med. Res..

[14] Fiseha T., Gebru T., Gutema H., Debela Y. (2015). Tuberculosis treatment outcome among HIV co-infected patients at Mizan-Aman general hospital, Southwest Ethiopia: a retrospective study. J. Bioeng Biomed. Sci..

[15] Kefale A.T., Anagaw Y.K. (2017). Outcome of tuberculosis treatment and its predictors among HIV infected patients in southwest Ethiopia. Int. J. Gen. Med..

[16] Madan C., Chopra K.K., Satyanarayana S., Surie D., Chadha V., Sachdeva K.S., Khanna A., Deshmukh R., Dutta L., Namdeo A. (2018). Developing a model to predict unfavourable treatment outcomes in patients with tuberculosis and human immunodeficiency virus co-infection in Delhi, India. PLoS One.

[17] Sileshi B., Deyessa N., Girma B., Melese M., Suarez P. (2013). Predictors of mortality among TB-HIV Co-infected patients being treated for tuberculosis in Northwest Ethiopia: a retrospective cohort study. BMC Infect. Dis..

[18] Sinshaw Y., Alemu S., Fekadu A., Gizachew M. (2017). Successful TB treatment outcome and its associated factors among TB/HIV co-infected patients attending Gondar University Referral Hospital, Northwest Ethiopia: an institution based cross-sectional study. BMC Infect. Dis..

[19] Beyene Y., Geresu B., Mulu A. (2016). Mortality among tuberculosis patients under DOTS programme: a historical cohort study. BMC Publ. Health.

[20] Eshetie S., Gizachew M., Alebel A., van Soolingen D. (2018). Tuberculosis treatment outcomes in Ethiopia from 2003 to 2016, and impact of HIV co-infection and prior drug exposure: a systematic review and meta-analysis. PLoS One.

[21] Ogyiri L., Lartey M., Ojewale O., Adjei A.A., Kwara A., Adanu R.M., Torpey K. (2019). Effect of HIV infection on TB treatment outcomes and time to mortality in two urban hospitals in Ghana-a retrospective cohort study. Pan African Medical Journal.

[22] Rocha M., Pereira S., Ferreira L., Barros H. (2003). The role of adherence in tuberculosis HIV‐positive patients treated in ambulatory regimen. Eur. Respir. J..

[23] Adebimpe W., Asekun-Olarinmoye E., Hassan A., Abodunrin O., Olarewaju S., Akindele A. (2011). Treatment outcomes among human immunodeficiency virus and tuberculosis co-infected pregnant women in resource poor settings of South-western Nigeria. Sierra Leone Journal of Biomedical Research.

[24] Corbett E.L., Watt C.J., Walker N., Maher D., Williams B.G., Raviglione M.C., Dye C. (2003). The growing burden of tuberculosis: global trends and interactions with the HIV epidemic. Arch. Intern. Med..

[25] Mekonnen D., Derbie A., Desalegn E. (2015). TB/HIV co-infections and associated factors among patients on directly observed treatment short course in Northeastern Ethiopia: a 4 years retrospective study. BMC Res. Notes.

[26] Warkari P.D., Nakel M.P., Mahajan S.M., Adchitre S.A. (2017). Study of treatment outcome of tuberculosis among HIV co-infected patients: a cross sectional study in Aurangabad city, Maharashtra. International Journal Of Community Medicine And Public Health.

[27] Tola A., Minshore K.M., Ayele Y., Mekuria A.N. (2019). Tuberculosis treatment outcomes and associated factors among TB patients attending public hospitals in Harar town, eastern Ethiopia: a five-year retrospective study. Tuberculosis research and treatment.

[28] Schutz C., Meintjes G., Almajid F., Wilkinson R.J., Pozniak A. (2010). Clinical management of tuberculosis and HIV-1 co-infection. Eur Respiratory Soc.

[29] Carlucci J.G., Blevins Peratikos M., Kipp A.M., Lindegren M.L., Du Q.T., Renner L., Reubenson G., Ssali J., Yotebieng M., Mandalakas A.M., Davies M.A. (2017). Tuberculosis treatment outcomes among HIV/TB co-infected children in the International Epidemiology Databases to Evaluate AIDS (IeDEA) network. J. Acquir. Immune Defic. Syndr..

[30] Tessema B., Muche A., Bekele A., Reissig D., Emmrich F., Sack U. (2009). Treatment outcome of tuberculosis patients at Gondar University Teaching Hospital, Northwest Ethiopia. A five-year retrospective study. BMC Publ. Health.

[31] Shastri S., Naik B., Shet A., Rewari B., De Costa A. (2013). TB treatment outcomes among TB-HIV co-infections in Karnataka, India: how do these compare with non-HIV tuberculosis outcomes in the province?. BMC Publ. Health.

[32] Asebe G., Dissasa H., Teklu T., Gebreegizeabhe G., Tafese K., Ameni G. (2015). Treatment outcome of tuberculosis patients at Gambella Hospital, Southwest Ethiopia: three-year retrospective study. Journal of Infectious Diseases & Therapy.

[33] Shaweno D., Worku A. (2012). Tuberculosis treatment survival of HIV positive TB patients on directly observed treatment short-course in Southern Ethiopia: a retrospective cohort study. BMC Res. Notes.

[34] Nglazi M.D., Bekker L.-G., Wood R., Kaplan R. (2015). The impact of HIV status and antiretroviral treatment on TB treatment outcomes of new tuberculosis patients attending co-located TB and ART services in South Africa: a retrospective cohort study. BMC Infect. Dis..

[35] Clérigo V., Mourato T., Gomes C., Castro A. (2018). Impact of HIV status, CD4 count and antiretroviral treatment on tuberculosis treatment outcomes in a low-burden country. J. Tubercul. Res..

[36] Tola A., Mishore K.M., Ayele Y., Mekuria A.N., Legese N. (2019). Treatment outcome of tuberculosis and associated factors among TB-HIV Co-infected patients at public hospitals of Harar town, eastern Ethiopia. A five-year retrospective study. BMC Publ. Health.

[37] Ahmed A., Mekonnen D., Shiferaw A.M., Belayneh F., Yenit M.K. (2018). Incidence and determinants of tuberculosis infection among adult patients with HIV attending HIV care in north-east Ethiopia: a retrospective cohort study. BMJ Open.

[38] Saini S., Singh M., Garg A. (2016). A retrospective cohort study of treatment outcome among HIV positive and HIV negative TB patients in Chandigarh, India. Indian J. Community Health.

[39] Gandhi N.R., Moll A.P., Lalloo U., Pawinski R., Zeller K., Moodley P., Meyer E., Friedland G. (2009). Successful integration of tuberculosis and HIV treatment in rural South Africa: the Sizonq'oba study. JAIDS Journal of Acquired Immune Deficiency Syndromes.

[40] Federal Ministry of Health of Ethiopia (FMOH) (July 2011 - June 2012). Federal Ministry of Health’ Preliminary Report of Ethiopia National TB/HIV Sentinel Surveillance. One Year Report.

[41] Akanbi K., Ajayi I., Fayemiwo S., Gidado S., Oladimeji A., Nsubuga P. (2019). Predictors of tuberculosis treatment success among HIV-TB co-infected patients attending major tuberculosis treatment sites in Abeokuta, Ogun State, Nigeria. The Pan African medical journal.

[42] Smith I. (2003). Mycobacterium tuberculosis pathogenesis and molecular determinants of virulence. Clin. Microbiol. Rev..

[43] Kwan C.K., Ernst J.D. (2011). HIV and tuberculosis: a deadly human syndemic. Clin. Microbiol. Rev..

[44] Ali S.A., Mavundla T.R., Fantu R., Awoke T. (2016). Outcomes of TB treatment in HIV co-infected TB patients in Ethiopia: a cross-sectional analytic study. BMC Infect. Dis..

[45] Kassa J., Dedefo M., Korsa A., Dibessa T. (2018). Factors affecting treatment outcome of tuberculosis among tuberculosis patients in West Ethiopia. J. Bioanal. Biomed..

[46] Ministry of Health of Ethiopia (MOH) (2013). Guidelines for Clinical and Programmatic Management of TB, TB/HIV and Leprosy.

[47] Ejeta E., Chala M., Arega G., Ayalsew K., Tesfaye L., Birhanu T., Disassa H. (2015). Outcome of tuberculosis patients under directly observed short course treatment in western Ethiopia. The Journal of Infection in Developing Countries.

[48] Worku S., Derbie A., Mekonnen D., Biadglegne F. (2018). Treatment outcomes of tuberculosis patients under directly observed treatment short-course at Debre Tabor General Hospital, northwest Ethiopia: nine-years retrospective study. Infectious Diseases of Poverty.

[49] Agha R., Abdall-Razak A., Crossley E., Dowlut N., Iosifidis C., Mathew G. (2019). STROCSS 2019 Guideline: strengthening the reporting of cohort studies in surgery. Int. J. Surg..

[50] Federal Ministry of Health of Ethiopia (FMOH) (2008). Federal Ministry of Health of Ethiopia: Manual of Tuberculosis, Leprosy and TB/HIV Prevention and Control Programme.

[51] research Registry (2020). researchregistry6188. https://www.researchregistry.com/browse-the%20registry#home/registrationdetails/5f9d14da5e2b290015631061/.

[52] World Health Organization, Stop TB Initiative (2010). Treatment of Tuberculosis: Guidelines.

[53] Kassu A., Mengistu G., Ayele B., Diro E., Mekonnen F., Ketema D., Moges F., Mesfin T., Getachew A., Ergicho B. (2007). Coinfection and clinical manifestations of tuberculosis in human immunodeficiency virus-infected and-uninfected adults at a teaching hospital, northwest Ethiopia. J. Microbiol. Immunol. Infect..

[54] Getahun B., Ameni G., Medhin G., Biadgilign S. (2013). Treatment outcome of tuberculosis patients under directly observed treatment in Addis Ababa, Ethiopia. Braz. J. Infect. Dis..

[55] Hamusse S.D., Demissie M., Teshome D., Lindtjørn B. (2014). Fifteen-year trend in treatment outcomes among patients with pulmonary smear-positive tuberculosis and its determinants in Arsi Zone, Central Ethiopia. Glob. Health Action.

[56] Melese A., Zeleke B., Ewnete B. (2016). Treatment outcome and associated factors among tuberculosis patients in Debre Tabor, Northwestern Ethiopia: a retrospective study. Tuberculosis research and treatment.

[57] Beza M.G., Wubie M.T., Teferi M.D., Getahun Y.S., Bogale S.M., Tefera S.B. (2013). A five years tuberculosis treatment outcome at Kolla Diba Health Center, Dembia District, Northwest Ethiopia: a retrospective cross-sectional analysis. J Infect Dis Ther.

[58] Abebe G., Bonsa Z., Kebede W. (2019). Treatment outcomes and associated factors in tuberculosis patients at Jimma University Medical Center: a 5-year retrospective study. International Journal of Mycobacteriology.

[59] Tweya H., Feldacker C., Phiri S., Ben-Smith A., Fenner L., Jahn A., Kalulu M., Weigel R., Kamba C., Banda R. (2013). Comparison of treatment outcomes of new smear-positive pulmonary tuberculosis patients by HIV and antiretroviral status in a TB/HIV clinic, Malawi. PLoS One.

[60] World Health Organization (2006). The Stop TB Strategy: Building on and Enhancing DOTS to Meet the TB-Related Millennium Development Goals.

[61] Raviglione M.C., Uplekar M.W. (2006). WHO's new Stop TB Strategy. Lancet.

[62] Uplekar M., Weil D., Lonnroth K., Jaramillo E., Lienhardt C., Dias H.M., Falzon D., Floyd K., Gargioni G., Getahun H. (2015). WHO's new end TB strategy. Lancet.

[63] Uplekar M., Raviglione M. (2015). WHO's End TB Strategy: from stopping to ending the global TB epidemic. Indian J. Tubercul..

[64] Shargie E.B., Lindtjørn B. (2005). DOTS improves treatment outcomes and service coverage for tuberculosis in South Ethiopia: a retrospective trend analysis. BMC Publ. Health.

[65] Ansa G.A., Walley J.D., Siddiqi K., Wei X. (2012). Assessing the impact of TB/HIV services integration on TB treatment outcomes and their relevance in TB/HIV monitoring in Ghana. Infectious Diseases of Poverty.

[66] Tripathy S., Anand A., Inamdar V., Manoj M., Khillare K., Datye A., Iyer R., Kanoj D., Thakar M., Kale V. (2011). Clinical response of newly diagnosed HIV seropositive & seronegative pulmonary tuberculosis patients with the RNTCP short course regimen in Pune, India. Indian J. Med. Res..

[67] World Health Organization (2011). Global Tuberculosis Report 2011. https://apps.who.int/iris/handle/10665/44728.

[68] World Health Organization (2014). Global Tuberculosis Report 2014. https://apps.who.int/iris/handle/10665/137094.

[69] World Health Organization (2017). Global Tuberculosis Report 2017. https://www.who.int/tb/publications/global_report/gtbr2017_main_text.pdf.

[70] Wen Y., Zhang Z., Li X., Xia D., Ma J., Dong Y., Zhang X. (2018). Treatment outcomes and factors affecting unsuccessful outcome among new pulmonary smear positive and negative tuberculosis patients in Anqing, China: a retrospective study. BMC Infect. Dis..

[71] Ananthakrishnan R., Kumar K., Ganesh M., Kumar A.M., Krishnan N., Swaminathan S., Edginton M., Arunagiri K., Gupta D. (2013). The profile and treatment outcomes of the older (aged 60 years and above) tuberculosis patients in Tamilnadu, South India. PLoS One.

[72] Karo B., Krause G., Hollo V., van der Werf M.J., Castell S., Hamouda O., Haas W. (2016). Impact of HIV infection on treatment outcome of tuberculosis in Europe. AIDS.

[73] Muñoz-Sellart M., Cuevas L., Tumato M., Merid Y., Yassin M. (2010). Factors associated with poor tuberculosis treatment outcome in the Southern Region of Ethiopia. Int. J. Tubercul. Lung Dis..

[74] Gelaw M., Genebo T., Dejene A., Lemma E., Eyob G. (2001). Attitude and social consequences of tuberculosis in Addis Ababa, Ethiopia. East Afr. Med. J..

[75] Sengul A., Akturk U.A., Aydemir Y., Kaya N., Kocak N.D., Tasolar F.T. (2015). Factors affecting successful treatment outcomes in pulmonary tuberculosis: a single-center experience in Turkey, 2005–2011. The Journal of Infection in Developing Countries.

[76] Ali M.K., Karanja S., Karama M. (2017). Factors associated with tuberculosis treatment outcomes among tuberculosis patients attending tuberculosis treatment centres in 2016-2017 in Mogadishu, Somalia. Pan African Medical Journal.

[77] Ramos J., Reyes F., Facin R., Tesfamariam A. (2008). Surgical lymph node biopsies in a rural Ethiopian hospital: histopathologic diagnoses and clinical characteristics. Ethiop. Med. J..

[78] Tekle B., Mariam D., Ali A. (2002). Defaulting from DOTS and its determinants in three districts of Arsi Zone in Ethiopia. Int. J. Tubercul. Lung Dis..

[79] Sanchez M., Bartholomay P., Arakaki-Sanchez D., Enarson D., Bissell K., Barreira D., Harries A., Kritski A. (2012). Outcomes of TB treatment by HIV status in national recording systems in Brazil, 2003–2008. PLoS One.

[80] Yone E.W.P., Kuaban C., Kengne A.P. (2012). HIV testing, HIV status and outcomes of treatment for tuberculosis in a major diagnosis and treatment centre in Yaounde, Cameroon: a retrospective cohort study. BMC Infect. Dis..

[81] Addis Z., Birhan W., Alemu A., Mulu A., Ayal G., Negash H. (2014). Treatment outcome of tuberculosis patients in Azezo health center, north West Ethiopia. Int. J. Curr. Sci. Res..

[82] Vijay S., Kumar P., Chauhan L.S., Rao S.V.N., Vaidyanathan P. (2011). Treatment outcome and mortality at one and half year follow-up of HIV infected TB patients under TB control programme in a district of South India. PLoS One.

[83] Ambadekar N., Zodpey S., Soni R., Lanjewar S. (2015). Treatment outcome and its attributes in TB-HIV co-infected patients registered under Revised National TB Control Program: a retrospective cohort analysis. Public health.

[84] Ruiz-Navarro M.D., Espinosa J.H., Hernández M.B., Franco A.D., Carrillo C.C., García A.D., Fulgueiras A.G., Diz P.G., De Valdivielso M.L., Fernández M.V. (2005). Effects of HIV status and other variables on the outcome of tuberculosis treatment in Spain.

[85] Falvo J.V., Ranjbar S., Jasenosky L.D., Goldfeld A.E. (2011). Arc of a vicious circle: pathways activated by Mycobacterium tuberculosis that target the HIV-1 long terminal repeat. American journal of respiratory cell and molecular biology.

